# Authority and Authorship: Examining Leadership's Role in Medical Publications

**DOI:** 10.1177/15562646251392340

**Published:** 2025-10-31

**Authors:** Vygintas Aliukonis, Eugenijus Gefenas

**Affiliations:** 1 Centre for Health Ethics, Law and History, Institute of Health Sciences, Faculty of Medicine, 54694 Vilnius University, Vilnius, Lithuania

**Keywords:** authorship, leadership, research integrity, medical publishing, bibliometrics, academic ethics

## Abstract

**Background:**

Academic medicine often struggles to balance leadership duties with maintaining research productivity. The impact of holding a position of power on authorship practices in a hierarchical environment remains underexplored. We addressed this gap by examining how leadership roles influence publication and collaboration patterns in Lithuanian medical academia.

**Methods:**

We performed a bibliometric analysis of ten-year publication records for 633 Lithuanian medical researchers in formal leadership positions (department heads, center directors, and similar formal roles), comparing their output and authorship patterns to those of peers without such roles. Publication data were collected from PubMed and a national academic library, capturing total publications, author order (first/middle/last author positions), and co-authorship counts. We used statistical tests to compare groups and applied the Gini coefficient to assess inequality in research output.

**Results:**

Leaders showed distinct authorship roles and collaboration patterns. Compared to equally productive non-leaders, leaders had significantly fewer first-authored papers (10.79% vs 36.31%) and more last-authored (36.42% vs 23.57%) and middle-authored contributions (52.78% vs 40.12%). Leaders published more papers (average 78.42 vs 49.41), in Web of Science–indexed journals (average 49.44 vs 27.68), and had higher h-indices (19.66 vs 12.59) (all *p* < 0.001). They also more frequently co-authored in larger teams (>5 co-authors: 58.76% vs 51.79%, *p* < 0.001). Output inequality among leaders was high (Gini = 0.718). Gender trends differed: prolific leaders were mostly men, while prolific non-leaders were mostly women. Importantly, these authorship patterns remained consistent across leader subgroups with varying productivity levels.

**Conclusions:**

Leadership position significantly impacts authorship practices and research productivity in Lithuanian medical academia -leaders display different patterns of collaboration and authorship positions, along with considerable institutional and gender disparities. The results illustrate how hierarchical power dynamics shape academic publishing in Lithuanian medical institutions. This evaluation could lead to important changes for organizational development and policies that ensure authorship credit accurately reflects actual contributions to research. From a research ethics perspective, authorship involves both accountability and recognition. The leadership-related shifts we observe require ethical scrutiny; our bibliometric analysis reveals structural patterns but cannot determine whether specific papers meet ICMJE authorship criteria.

## Introduction

In the competitive and collaborative world of academic research, authorship is more than just a marker of contribution; it is a gateway to recognition, funding, and career advancement. However, the path to securing authorship is fraught with challenges, where various influences intersect, obscuring clear lines of contribution. In hierarchical academic settings, individuals in power may receive authorship credit regardless of their actual contribution – a practice known as honorary authorship, which raises concerns about authorship integrity. Prior research indicates that individuals in a position of power are more frequently included as honorary authors. For instance, pooled survey data suggest that 20% of researchers report automatically listing senior members, such as department heads, as authors (Meursinge Reynders et al., 2024). This practice sometimes increases to 25% or more, depending on the discipline (Kressel & Dixon, 2011). Findings like these usually come from anonymous surveys.

**Figure 1. fig1-15562646251392340:**
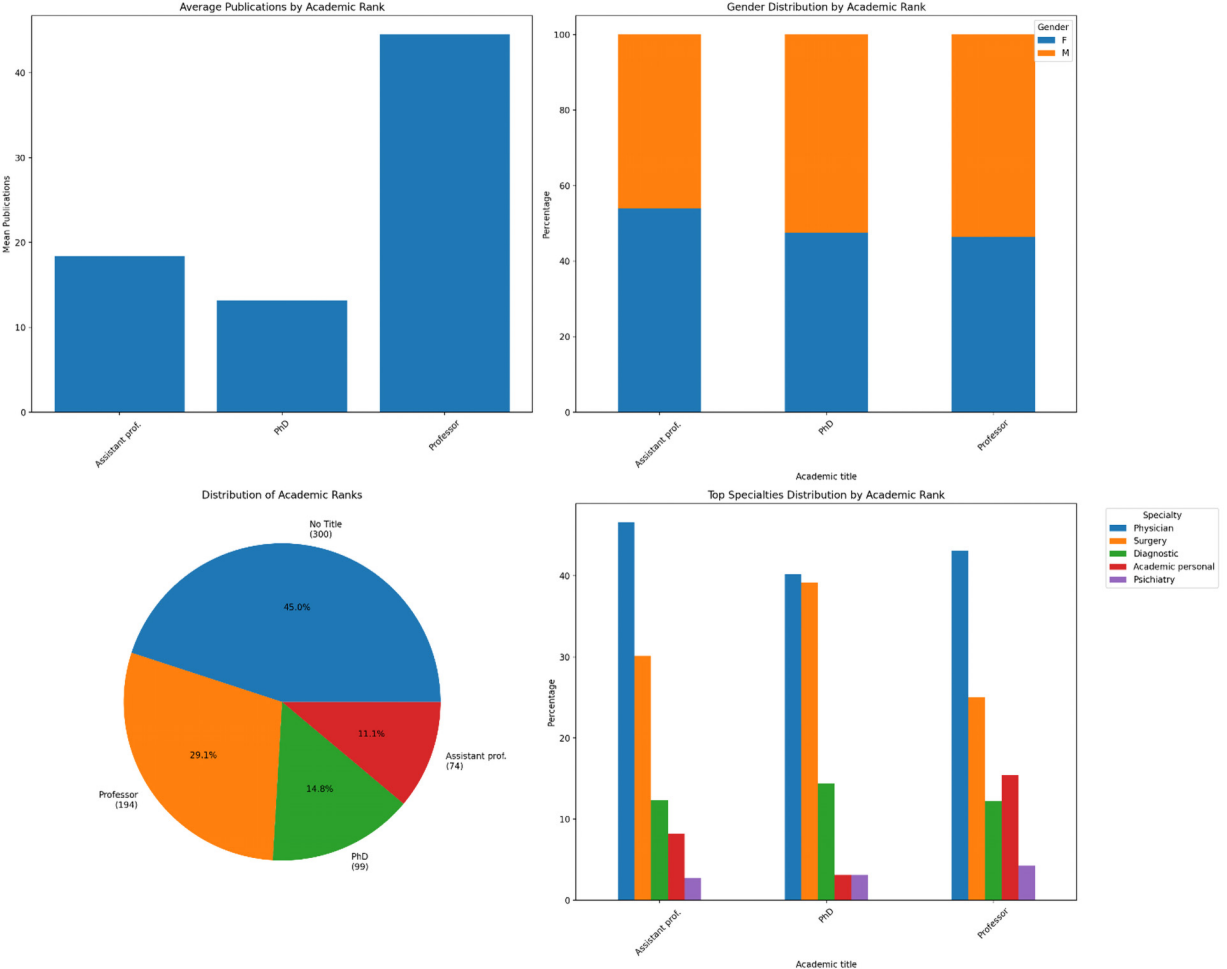
General statistics.

**Figure 2. fig2-15562646251392340:**
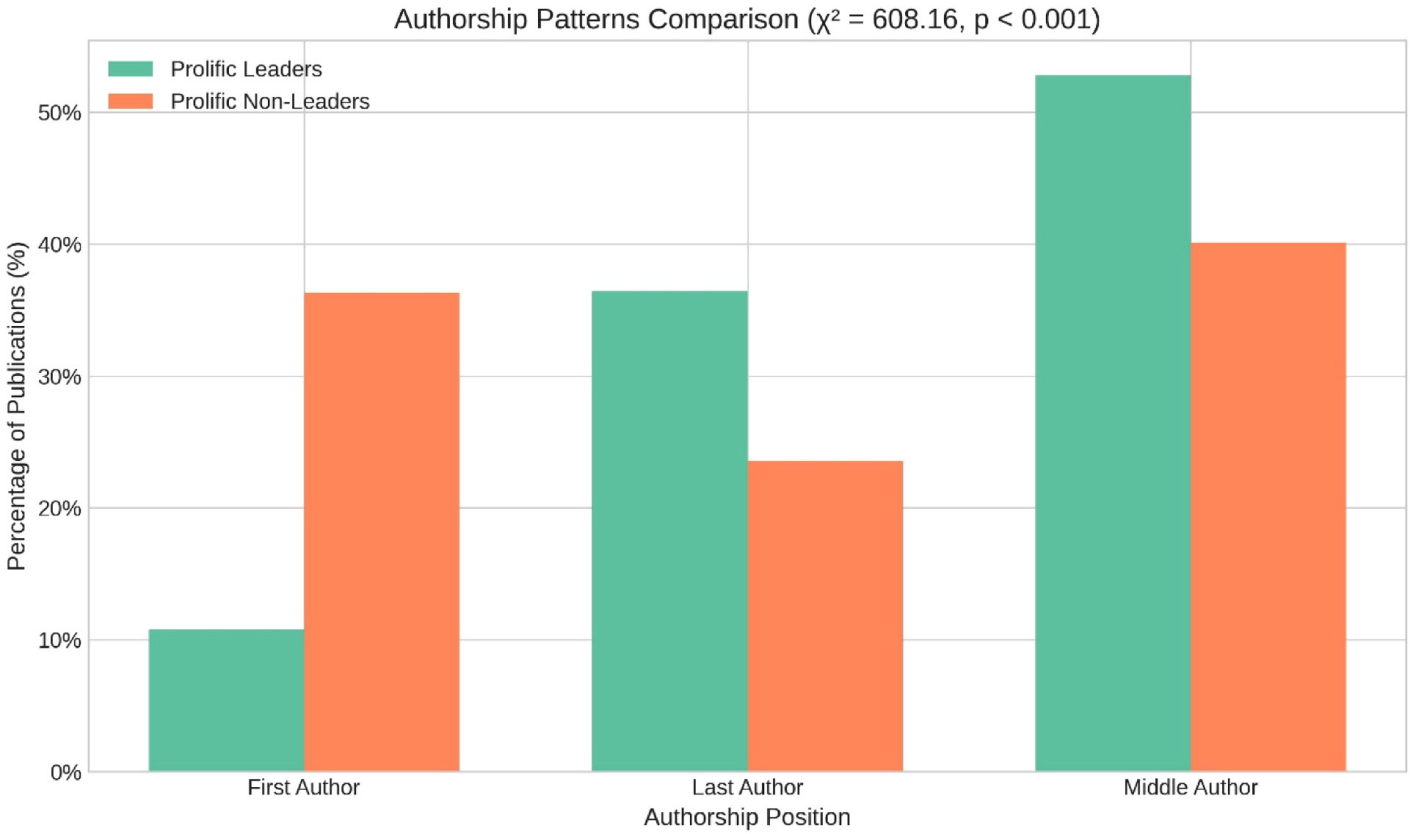
Authorship patterns comparison.

**Figure 3. fig3-15562646251392340:**
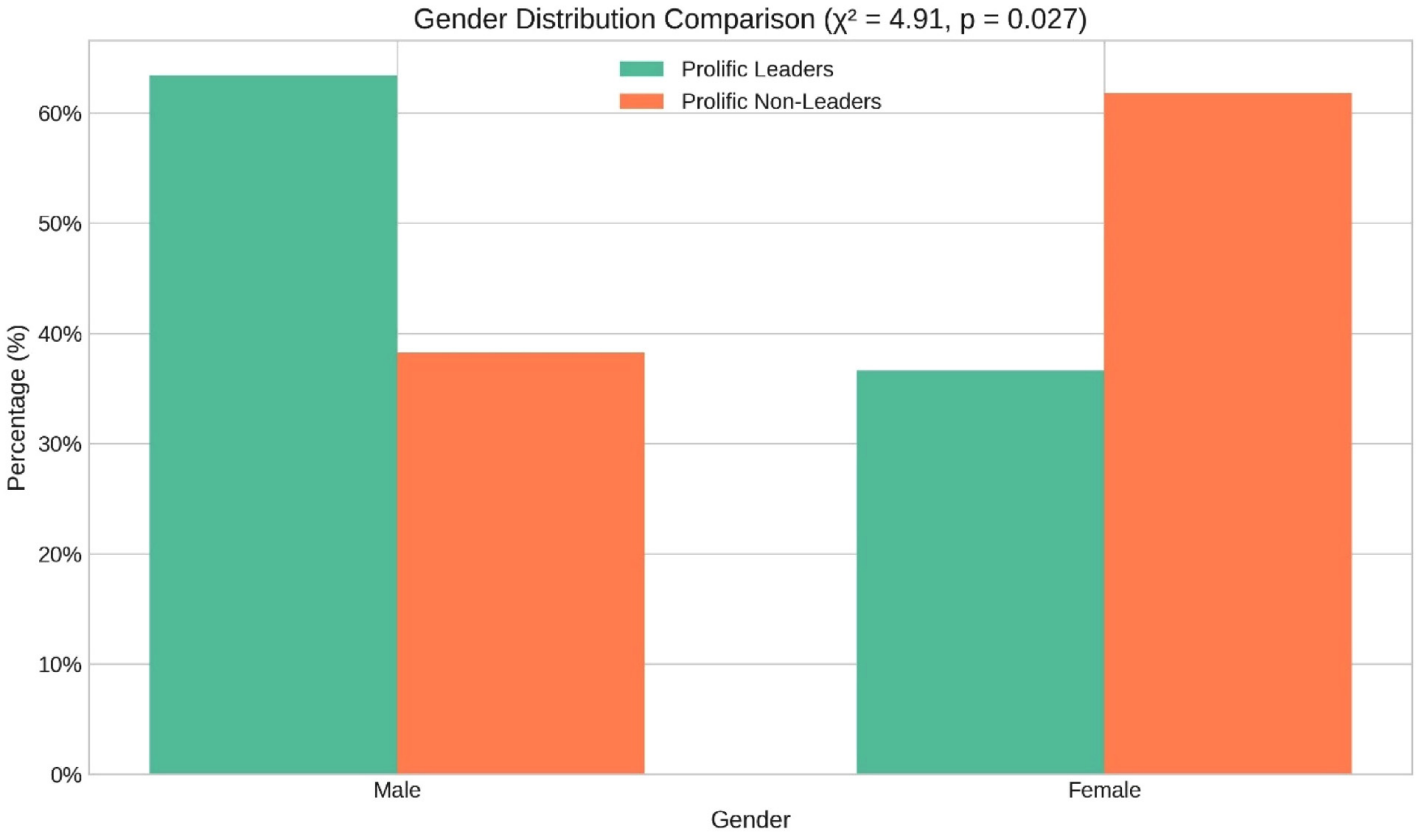
Gender distribution comparison.

**Figure 4. fig4-15562646251392340:**
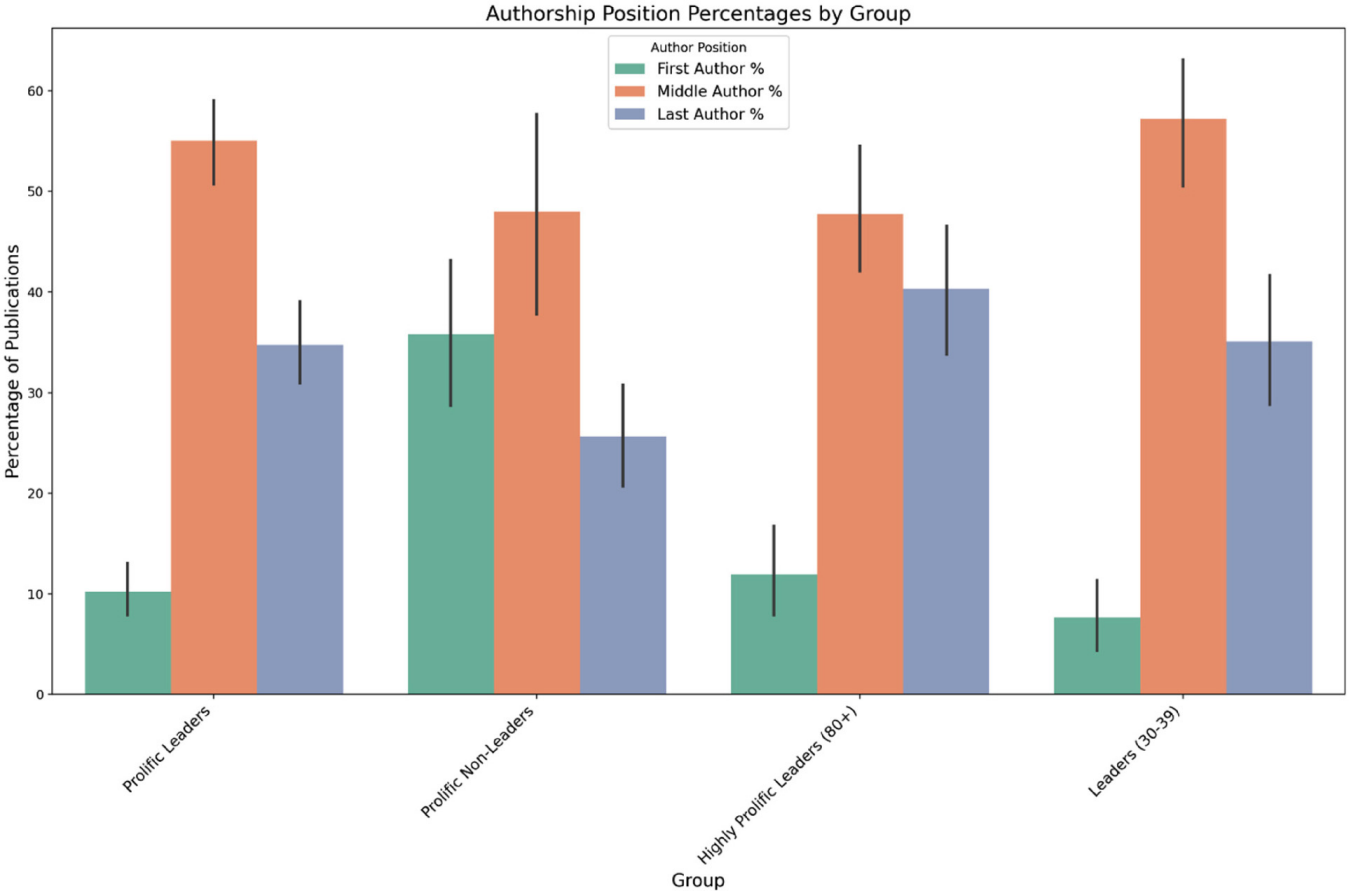
Authorship position percentages by group.

Although measuring the spread of honorary authorship is an important objective, the aim of our study has a different focus – exploring the impact of power position on broader patterns of researchers’ interrelationships relevant to authorship. These authorship patterns raise a clear concern about research ethics. Giving publication credit to individuals who contributed little or nothing is unfair to those who did the work and damages the integrity and transparency of the scientific record. It also misattributes credit and breaches principles of fairness in research. This ethical issue sets the stage for our study's focus on leadership and authorship. This study relies on bibliometric data, which helps reveal authorship patterns among researchers and serves as a tool for profiling them based on those patterns, including their position on the list of authors, the number of co-authors, and the total number of publications. Bibliometric data has already been used to assess patterns of authorship. For instance, using bibliometric data, Ponzi (2020) demonstrated that higher-ranking academics had significantly higher publication rates, with output increasing dramatically as one climbs the academic ladder. Although Ponzi's study did not explicitly examine honorary authorship, his findings suggest a ‘pyramid scheme’ effect in science: those in positions of power tend to accumulate disproportionately large publication counts. In line with this pattern, Gureyev (Mazov & Gureev, n.d.) used a bibliometric approach to calculate the scientific output of authors in different stages of their career and analyzed how gaining more influence in the academic world (either by defending their thesis or obtaining the position of power in an institution) reflects on the total count of publications. He claims that the patterns he discovered indicate honorary authorship. Others, like Hart and Perlis (2021), used other bibliometric tools, like the Gini coefficient, to highlight inequalities in authorship, demonstrating that a small group of prolific authors dominate high-impact publications.[Fig fig1-15562646251392340][Fig fig2-15562646251392340][Fig fig3-15562646251392340][Fig fig4-15562646251392340]

Consistent with evidence that authorship order signals contribution (Marušić et al., 2011), studies also shows that seniority is linked to authorship order: professors and senior researchers are less often first authors and more often last authors (Costas & Bordons, 2011). Moreover, authorship credit in medical journals has become increasingly concentrated among fewer individuals in high-impact publications (Hart & Perlis, 2021). Third, team science has grown, with larger author lists and evolving collaboration structures that influence how credit is allocated (Tscharntke et al., 2007). Fourth, concerns about honorary or automatic senior authorship are well documented, including a recent systematic review and meta-analysis on automatically listing senior departmental members as co-authors in the health sciences, and surveys in leading medical journals reporting honorary/ghost authorship (Meursinge Reynders et al., 2024). Against this context, our study isolates the role of formal institutional leadership—distinct from general seniority—by comparing equally senior leaders with equally senior non-leaders and by examining multiple indicators (author order, team size, inequality). This distinction is ethically significant: authorship should reflect contribution, not institutional role. Readers rely on author order as a quick signal of roles; if rank dictates placement, that signal is unreliable. When status drives inclusion, who designed the study, ran the analyses, or wrote the paper becomes unclear, and post-publication questions and corrections become harder. By isolating leadership effects, we highlight settings where clearer contributorship statements would add value.[Table table1-15562646251392340][Table table2-15562646251392340][Table table3-15562646251392340]

**Table 1. table1-15562646251392340:** Publication Output, Gender Distribution, and Academic Rank.

	Publications (Mean ± std)	Gender Distribution	Academic Rank
Prolific non-leaders	49.4 ± 23.4	M: 13 (38.2%), F: 21 (61.8%)	Professor (100.0%)
Prolific leaders	78.4 ± 40.9	M: 45 (63.4%), F: 26 (36.6%)	Professor (84.5%), PhD (8.5%), Assistant prof. (5.6%)
Non-prolific leaders	8.1 ± 10.5	M: 254 (45.2%), F: 308 (54.8%)	Professor (17.8%), PhD (16.5%), Assistant prof. (12.5%)
All leaders	16.0 ± 27.8	M: 299 (47.2%), F: 334 (52.8%)	Professor (25.3%), PhD (15.6%), Assistant prof. (11.7%)
All combined	17.7 ± 28.6	M: 312 (46.8%), F: 355 (53.2%)	Professor (29.1%), PhD (14.8%), Assistant prof. (11.1%)

**Table 2. table2-15562646251392340:** Specialties Distribution.

	Specialties
Prolific non-leaders	Physician (35.3%), Diagnostic (32.4%), Surgery (20.6%), Psychiatry (5.9%), Academic personnel (5.9%)
Prolific leaders	Physician (46.5%), Surgery (22.5%), Academic personnel (19.7%), Diagnostic (9.9%), Psychiatry (1.4%)
Non-prolific leaders	Physician (47.0%), Surgery (26.7%), Diagnostic (10.3%), Psychiatry (7.3%), Academic personnel (5.9%), Odontology (2.8%)
All leaders	Physician (46.9%), Surgery (26.2%), Diagnostic (10.3%), Academic personal (7.4%), Psychiatry (6.6%), Odontology (2.5%)
All combined	Physician (46.3%), Surgery (25.9%), Diagnostic (11.4%), Academic personal (7.3%), Psychiatry (6.6%), Odontology (2.4%)

**Table 3. table3-15562646251392340:** General Statistics for Authors.

Characteristic	Prolific Leaders	Prolific Non-Leaders	Statistical Test
Publication Type			
First author (%)	10.79%	36.31%	*p* < 0.001
Last author (%)	36.42%	23.57%	*p* < 0.001
Middle author (%)	52.78%	40.12%	*p* < 0.001
Co-authorship Pattern			
More than 5 co-authors (%)	58.76%	51.79%	*p* < 0.001
Five or fewer co-authors (%)	41.24%	48.21%	*p* < 0.001
Publication Metrics			
Mean publication count	78.42 ± 40.89	49.41 ± 23.41	*p* < 0.001
Mean H-index	19.66 ± 7.99	12.59 ± 8.11	*p* < 0.001

Despite these examples, we agree with Haustein and Larivière (2015), who, in their comprehensive analysis “The Use of Bibliometrics for Assessing Research” state that “*Most importantly, entities, such as researchers and institutions, should not be ranked by one indicator, but multiple metrics should be applied to mirror the complexity of scholarly communication*.” Thus, compared to prior studies that typically focus on a single metric or limited variable, our research integrates multiple bibliometric indicators to capture a more comprehensive view of authorship patterns among authors with positions of power, offering a richer understanding of academic authorship. For this study, ‘position of power’ is defined as holding formal leadership roles within academic medical institutions, such as department heads, center directors, or similar administrative positions. The study also examines gender disparities in leadership roles, their implications for publication practices, and inequalities in authorship and research output using the Gini coefficient for systemic disparities.

To interpret these patterns, we draw on two complementary ideas: role expectations and power–dependence within teams. Formal leadership can shift role expectations and collaboration patterns. Role theory holds that behavior tends to follow position-linked norms, so leaders more often coordinate and supervise rather than act as primary contributors (Anglin et al., 2022). Power–dependence theory adds that control over valued resources, such as lab space, funding, or career opportunities can influence how credit is shared and allocated within teams (Emerson, 1962). In hierarchical academic settings, these dynamics predict that leaders will appear more often as last- or middle-authors and less often as first-authors. This can misattribute credit and create confusion about who is responsible for what. These mechanisms are ethically important because they can disconnect credit from contribution and make responsibility less transparent. Guided by these ideas and prior evidence on seniority-related authorship shifts, we hypothesized that, relative to equally senior but non-leader peers, leaders would (H1) appear less often as first authors and more often as last or middle authors; (H2) co-author more frequently on larger teams; and (H3) display greater inequality of outputs within the leadership group.

## Methodology

### Data Collection

All individuals holding institutional positions of power within the sampled institutions were identified based on information available on the official websites of these institutions. In cases where individuals held multiple roles (e.g., head of a hospital center and chair of a department related to the same pathology), only one affiliation of the author was included in the study.

Given our hypothesis that holding a leadership position may significantly influence bibliographic profiles, our primary comparative analysis focused on two groups: prolific leaders and prolific non-leaders, which comprise two different groups of highly productive medical authors with similar academic ranks. Specifically, authors in leadership positions might demonstrate distinct patterns, such as the frequency of appearing in middle or last-author roles or having different collaborative networks (like having a higher count of co-authors). Any observed differences between groups would thus highlight the potential impact of institutional authority on authorship practices. We selected groups with prolific authors because a sufficient volume of data points is necessary for performing meaningful statistical analyses. Without enough data, some differences may be obscured by random variation, limiting the reliability of conclusions. Our study defined prolific authors as those who published over 40 articles over 10 years. The entire group of leaders was used only for descriptive statistics. Furthermore, to explore whether an increase in publication count is associated with shifts in bibliographic profiles, we distinguished between two subgroups within the prolific leaders’ groups —less prolific leaders (30–39 publications per 10 years) and very prolific leaders (more than 80 publications per 10 years). These subgroups are used to determine if changes in publication counts bring a leader's bibliographic profile closer or further to that of non-leaders, thereby shedding light on whether publication productivity or the mere status of holding a leadership role plays a more decisive role in authorship patterns.

Publications were sourced from the PubMed database and the Lithuanian Academic Electronic Library (eLABa). eLABa includes English-language articles and many Lithuanian articles that are not indexed in PubMed. The analysis focused solely on journal articles, excluding letters to the editor, conference materials, textbooks, and similar publications.

A single researcher collected data. Author gender was researcher-interpreted from publicly available institutional profiles (e.g., given names, stated pronouns, photographs. In ambiguous cases, multiple public sources (e.g., university news pages and professional bios were cross-checked before assigning gender.

All authors were manually verified to avoid issues of homonymy (Costas & Bordons, 2011; Pruschak & Hopp, 2022).

For all authors, we collected data on several variables like authors’ gender, workplace, and academic title (rank). The title was recorded and categorized as professor, assistant professor, or PhD (including only those who had defended a thesis). We gathered data on authors’ specialties to analyze differences in publication trends across various fields. The total number of publications over the past decade served as a primary measure of research productivity.

To assess collaboration levels and network size among authors, we calculated the average number of co-authors per publication and publication count with different numbers of co-authors - more than 5, 10, or 20 co-authors. We also gathered data for authorship positions, which were analyzed by counting the number of publications where the individual was the first, last, or middle author.

### Statistical Analysis

We used several statistical methods in this study. To summarize authorship metrics, we applied descriptive statistics and conducted comparative analyses with independent samples t-tests and Mann-Whitney U tests to identify differences in key bibliometric indicators between prolific leaders and non-leaders. Multiple linear and logistic regression models were used to adjust for potential confounding variables. Finally, the Gini coefficient was calculated to measure inequalities in publication output and authorship roles.

## Explanation for Variables Used

### Number of Publications

A large-scale study by Norwegian researchers demonstrated that the average production of publications per author per year in the biomedical field is less than one. They also showed that professors are the most prolific individuals regardless of field, gender, or age, and as academic rank decreases, so does the number of publications (Rørstad & Aksnes, 2015). Some research suggests that exceptionally high publication counts may sometimes be associated with practices such as honorary authorship (Dang et al., 2015). In our study, the cutoff point is arbitrary, as authors with scientific output of 40 or more publications are not hyper prolific and might be normal in academia. However, publishing four times more often than average while leading departments raises reasonable doubts about the scientific integrity of those authors. Other researchers have similarly used the total number of articles as a key metric in bibliometric studies on authorship in many different academic fields. Their findings demonstrate a correlation between a higher publication count and honorary authorship (Meho & Akl, 2024).

Although previous studies have examined publication patterns across various disciplines—and some have even compared different medical specialties (Dang et al., 2015; Rørstad & Aksnes, 2015) —few have established definitive numerical thresholds for publications per author that could serve as markers of questionable research practices. We acknowledge that the number of publications can vary considerably; therefore, any threshold used to identify potential irregularities must be interpreted on a case-by-case basis.

### Number of Co-Authors

In addition to the number of publications, the number of co-authors is another commonly measured bibliometric variable. A recent extensive study has revealed that the average number of authors per publication has increased by over one and a half times in the past 20 years, rising from 3.99 to 6.25. Moreover, the number of authors per publication varies significantly across different medical specialties. A study evaluating 23 medical specialties between 2005 and 2017 reported that original research articles experienced the greatest increase in authorship, with a different average number of authors added per article across specialties (An et al., 2020). While specific fields, like clinical trials, inherently involve larger collaborative teams, studies still found a noticeable increase in the number of coauthors included in clinical trials over the past two decades (Jakab et al., 2024). This expansion in the number of authors has raised concerns about the potential for honorary authorship.

While the growing complexity of scientific research sometimes justifies the increasing number of co-authors, scholars investigating this phenomenon argue that the number of authors continues to grow at a rate unexplainable by the complexity of biomedical research (Hart & Perlis, 2021). Counting co-authorship reflects the degree of research collaboration and may serve as a proxy for detecting questionable authorship practices. For example, Dang et al. (2015) analyzed radiology journals and noted that ‘many articles with more than five co-authors include contributors whose actual input is minimal’. Some authors take an even stricter stance, stating that the more co-authors there are, the higher the likelihood of honorary authors being among them (Kandil, 2022; Villar, 2019). This finding suggests that a high number of co-authors may sometimes indicate the inclusion of honorary authors. Similarly, Larivière et al. (Fanelli & Larivière, 2016) found that team sizes have increased significantly over the past century, a trend that reflects enhanced collaboration but can also dilute individual contributions. Additionally, Wuchty et al. (2007) demonstrated that while larger teams tend to produce research with higher impact, the growth in co-authorship raises concerns about accountability. These studies illustrate why co-authorship should be measured alongside total publication output, as it offers nuanced insights into collaborative practices.

### Order of Authors

It is important to know not only how many articles authors publish and with whom they collaborate, but also the order in which their names appear on publications. Some studies indicate that meeting the ICJME criteria for authorship is heavily influenced by the order of authors, with the highest fulfillment for the first author and less for all others (Marušić et al., 2011). In biomedicine, research professors typically exhibit a very low percentage of first-authored papers. Although less straightforward to interpret, the middle author position often reflects substantial collaborative input that may be essential yet less clearly defined. For example, Tscharntke et al. (2007) noted that first authors usually fulfill the ICJME criteria for substantial contribution, whereas the contributions of middle authors can be more variable and less easily quantified. Moreover, Marušić et al. (2011) reported that the variability in contributions among middle authors can complicate accountability and may mask instances of honorary authorship.

## Results

### General Descriptive Statistics

We examined whether there is a meaningful association between an author's area of specialization and their authorship patterns (Tables 1, 2; Figure 1).

The results showed a strong link between specialization and authorship position (χ² ≈ 99.05, *p* < 0.001). Some specific cases were notable: psychiatry and Surgery had more first authorships than expected, while physicians had fewer first authorships but more last authorships. Despite these differences, the overall impact was small, indicating that specialization does not significantly affect authorship roles in this dataset.

However, the Gini coefficient indicates very significant disparities in publication output within specialties. Specifically, the surgery and physician specialties exhibit high inequality (Gini ∼0.70 and ∼0.74), suggesting that a limited number of authors contribute to a substantial portion of publications in these fields. In contrast, specialties such as psychiatry and academic personnel show lower inequality (Gini approximately 0.58 and 0.57), reflecting a somewhat more equal distribution of publications among authors in those areas. Regarding academic titles, authors holding the title of Professor had a Gini coefficient of approximately 0.40, indicating a moderate-to-high inequality in publication output within this group. This suggests that even among highly ranked academics, a subset of individuals publishes significantly more than their peers.

### Group Comparison

As we hypothesize that significant differences exist in publication counts, authorship patterns, collaboration networks, and research impact between prolific authors holding leadership positions and those who do not, we compared different variables across the two groups.

Comparison between prolific leaders and prolific non-leaders showed that prolific leaders had a higher mean total number of publications at 78.42 compared to 49.41 for prolific non-leaders (*p* < 0.001) (Table 3). They also published more in Web of Science (WOS) indexed journals, with a mean of 49.44 versus 27.68 (*p* < 0.001). Also, prolific leaders demonstrated a higher mean H-index of 19.66 compared to 12.59 for prolific non-leaders (*p* < 0.001).

Authorship patterns differed notably. Leaders were significantly less likely to be first authors compared to non-leaders (10.79% vs 36.31%), while being more likely to appear as last authors (35.42% vs 23.57%). This pattern suggests a shift in publication strategy as academics move into leadership positions, transitioning from primary researcher roles to supervisory and mentorship roles.

There was no significant difference in the average number of co-authors per publication between the groups (*p* = 0.590) (Figure 2). However, to determine whether prolific authors in leadership positions have a higher proportion of multi-authored papers, we compared the number of papers with more than five co-authors against those with five or fewer co-authors within each group. Leaders had 58.76%, publications with 5 or more coauthors while non leaders 51.79% (*p* < 0.001). These findings suggest that prolific authors in leadership positions are more likely to publish papers with a larger number of co-authors than those without leadership roles.

Regarding *academic rank*, prolific non-leaders consisted entirely of professors, which aligns with our expectations. This control group was specifically designed to include individuals with higher academic productivity, naturally correlating with their advanced academic titles. In contrast, the group of prolific leaders was predominantly composed of professors (84.5%), but also included individuals with lower academic ranks, such as PhDs (8.5%) and assistant professors (5.6%). Non-prolific leaders had a more mixed distribution of academic ranks, with a significantly lower proportion of professors.

Regarding *specialties*, prolific leaders were more often physicians and academic personnel compared to prolific non-leaders. Notably, almost one-fifth of prolific leaders were academic personnel, compared to only 5.9% among prolific non-leaders. Prolific non-leaders had a broader distribution across specialties.

#### Gender Analysis

Finally, our analysis revealed notable gender disparities across the groups. Prolific leaders were predominantly male (63%), whereas prolific non-leaders were mostly female (62%). This raises questions about broader structural factors in academia and medicine. Interestingly, women were slightly over-represented among the overall pool of leaders (when considering all 633 leaders, ∼53% were female). This nuance suggests that while women hold many leadership positions in absolute terms, the most prolific leaders tend to be men. Thus, the gender effect may be more about how leadership and productivity intersect: male leaders appear to contribute in different authorship patterns than female leaders. The slight surplus of women among all leaders counteracts a simple narrative of systemic exclusion – at least in this medical context – and points to a need to explore how gender and power together influence research roles, rather than gender alone (Figure 3).

#### Subgroup Analysis

To further evaluate the impact of leadership roles on bibliometric profiles, we extended the analysis to include four distinct groups: Prolific Leaders, Prolific Non-Leaders, Highly Prolific Leaders (those having 80+ publications), and Leaders with fewer publications (those having 30–39 publications). This comparison helps answer whether a leadership role alone influences the bibliometric profile or if publication volume is also a factor.

The analysis showed statistically significant differences between the groups in the middle author position. With prolific leaders occupying 52.78% and prolific non-leaders occupying 40.12%. Further comparisons between groups didn't show statistically significant results.

For the last author position percentages, there was a statistically significant difference with Highly Prolific Leaders (80+) and prolific leaders groups having higher percentages than Prolific Non-Leaders.

Examining the first author position percentages, we see that differences between all three leader groups and Prolific Non-Leaders were statistically significant for all comparisons. In contrast, the differences among the three leader groups were not significant. This suggests that people in leadership positions are less likely to be first authors compared to non-leaders, regardless of their publication volume (Figure 4).

Regarding collaborative patterns, the analysis showed no statistically significant differences between the groups in the average co-author count (*p* = 0.54). Also all groups showed similar percentages of publications with large teams (>5 co-authors), ranging from 51.8% to 60.6%, with statistically significant differences between prolific leaders and non prolific leaders groups.

## Discussion

It is not unusual for researchers to have fewer first-author publications and more last-author publications as their professional rank increases, often associated with seniority and project oversight. This effect has already been observed previously (Costas & Bordons, 2011). In the field of biomedicine, research professors typically exhibit a very low percentage of first-authored documents—often below 10%—and a high percentage of last-authored documents, approximately 50% (Costas & Bordons, 2011). Among highly prolific authors, last-author positions account for 42.5%, while first-author positions make up only 7.1% (Ioannidis et al., 2018).

While studies highlight the automatic inclusion of leaders in authorship roles without considering their actual contributions (Mazov & Gureev, n.d.), usually they do not distinguish between institutional leadership and roles like supervising students (Dang et al., 2015), which are common for professors. Our results reinforce what the literature shows about seniority and author position. However, unlike earlier studies, we examined two equally senior groups that differ only in formal leadership status.

We have also identified three distinct authorship patterns: a primary contributor—typically seen in researchers with a high proportion of first-author publications—indicates direct involvement in the research process. A team member pattern, usually represented by a middle author, reflects collaborative contribution, while a supervisory pattern, characterized by the mostly last author position, suggests a role in guiding and coordinating research projects. These profiles can be used to study authorship trends not only for individual authors but also for comparative studies across different groups (e.g., leaders versus non-leaders), thereby providing insights for refining authorship criteria and enhancing research integrity.

Prolific non-leaders are typically more often involved in the research and writing process – with roughly 36% as first-author contributions, and the remainder split between middle (≈40%) and last (≈24%) author roles. This profile suggests hands-on engagement, as they often lead or significantly contribute to the research (reflected by the high first-author rate). In contrast, prolific leaders tend to adopt a coordinating or supervisory role. The vast majority of their publications list them as middle authors (∼52%) or last authors (∼36%) and only about 10% of their papers are first-authored. This indicates that their primary role is guiding and overseeing research done by others, rather than engaging in hands-on work or drafting manuscripts. Less prolific leaders authorship profile showed a similar pattern: on average only ∼7% of their papers had them as first author, while about 61% were as middle author and 32% as last author. Although a few individuals in this group have a slightly higher direct contribution, the overall trend still reflects a predominantly supervisory role. Notably, this pattern occurs despite these individuals having more modest publication counts.

However, we also observed several instances where leaders with over 50 publications had fewer than 5% of them as first authors and a distinct group of 22 academic leaders (representing 21.2% of combined sample of prolific leaders and 30–39 group) who, despite having substantial publication records (averaging 39.1 publications each), have never appeared as first authors. These researchers demonstrate a predominantly team member pattern with an average of 36.3% of their publications in the last author position, and the remaining 63.7% in middle author positions, suggesting collaborative roles. Their publication patterns show a strong tendency toward team science, with 67.4% of their work involving large teams (more than 5 authors) and 19.1% involving very large teams (more than 10 authors). This extreme imbalance, combined with their position of authority, raises a potential red flag for honorary authorship. While such patterns do not necessarily prove wrongdoing by any individual, they suggest that holding a position of power may facilitate the receipt of authorship credit. However, senior investigators often shift from primary contributor roles to supervisory positions, which is shown in last-author and middle-author roles. Figuring out if authorship criteria were met would need qualitative follow-up—such as interviews with co-authors and looking at contributorship statements—which is beyond this study's scope.

Finally, according to our data, individuals in positions of power are more similar to each other than to the control group, regardless of the number of publications. Considering that our control group held high academic position, our findings reinforce the idea that authorship behavior is driven more by one's institutional role of power than academic rank or simply by productivity. Our analysis also revealed a strong correlation between total publications, number of multi-authored papers, and middle-author positions (*r* = 0.74–0.80), suggesting that these factors are interrelated in the authorship pattern characteristic of leaders. In other words, being in a position of power often coincides with contributing in a supervisory capacity rather than as a primary contributor. These findings show that bibliometric analysis can provide quantitative indicators that flag possible honorary authorship patterns. A leader's profile of many publications, few first-authorships, and frequent middle-authorships in large collaborations might serve as a warning sign, warranting closer examination of contribution levels.

## Conclusions

This study demonstrates differences in authorship patterns among medical researchers in Lithuania, depending on their leadership roles. Prolific researchers without leadership roles more often appear as first authors. This pattern reflects their active involvement in conducting research and manuscript preparation. Researchers in positions of power—both highly prolific and moderately prolific usually appear as middle or last authors. This coordinating authorship pattern indicates that their roles are more supervisory, guiding, or overseeing the research rather than directly conducting it.

The analysis revealed significant disparities even among leaders, with a Gini coefficient of 0.718 indicating substantial inequality in research productivity. Significant gender disparities were observed: men predominantly held leadership roles, while women were more frequently found in non-leadership positions. These results highlight the necessity for further exploration of structural inequalities and the broader implications of leadership on academic careers.

This research shows how different levels of authority in institutions affect publication patterns. While none of the findings are necessarily unethical, the observed trends are concerning. We urge increased awareness and further investigation into authorship practices within hierarchical academic structures. Ensuring that authorship credit accurately reflects contributions is necessary for research integrity, and our analysis provides a data-driven signal that this principle may be at risk in the current system.

We believe bibliometric data holds good promise for clarifying authorship practices and their relation to academic professional roles. By employing bibliometric analyses, we can better differentiate distinct author profiles based on their publishing patterns. These methods allow institutions to clearly distinguish researchers actively involved in conducting and writing research from those primarily engaged in supervisory or coordinating roles. Finally, such bibliometric profiling could reveal how these authorship roles evolve throughout a researcher's career and help institutions develop guidelines to support fairer and more accurate assessments of individual contributions, strengthening research integrity practices in academia. These findings align with the study's main goal: to show how leadership roles influence authorship practices in Lithuanian medical academia. It would be valuable to compare these results with data from other countries to determine if similar patterns exist internationally. Such comparative analyses could provide deeper insights into how cultural, institutional, and policy differences influence authorship behaviors and the role of leadership in academic publishing.

## Limitations

This study acknowledges several limitations that warrant consideration. Firstly, the focus on prolific authors affiliated with Lithuanian medical institutions may restrict the applicability of the findings to other contexts or academic disciplines. Furthermore, the cross-sectional design provides a snapshot of publication activities over a ten-year period, without accounting for temporal variations in publication practices or individual career progression. Additionally, the study did not evaluate continuity or thematic consistency of research topics within authors’ publication records, which could provide additional insights into authorship practices. Since a single coder performed data extraction and gender inference, a small risk of misclassification remains, particularly for gender classification.

## Educational Implications

Education on publication ethics is necessary at multiple levels of the academy as part of research ethics (fair credit and clear responsibility). Institutional leaders should regularly receive update sessions that recap the formal criteria for authorship and explicitly highlight that merely holding a leadership position does not justify co-authorship. In contrast, early- and mid-career researchers need training that empowers them to apply the same criteria with confidence and to recognize that they are under no obligation to list a supervisor who has not met the requirement for authorship, documenting contribution agreements at the start of a project can support fair credit and accountability.

At the institutional level, tracking authorship patterns can form part of proportionate ethics oversight. For instance, if an author suddenly transitions from being the first author on papers to primarily being a middle or last author after assuming a leadership role, it does not definitively indicate wrongdoing, but it does suggest the need for closer scrutiny. Having brief contribution notes or internal attestations could be a safeguard to protect the fairness of credit and the traceability of responsibility while avoiding presumptions of misconduct. This is a straightforward methodology for identifying potential questionable research practices at a level beyond the individual.

The following ‘Best Practices’ operationalize these educational aims into routine procedures for teams and departments.

## Best Practices

To implement these insights— and integrate the educational actions above— institutions should (1) require clear contribution statements accompanied by brief, role-specific training on how to use contributorship (e.g., CRediT) forms, (2) base authorship eligibility on demonstrated scholarly work rather than job titles, and (3) develop mentorship guidelines that encourage open discussions about author order early in a project and include these discussions in regular induction or training sessions for leaders and early-career researchers. Research leaders should demonstrate ethical behavior by limiting their own byline appearances to cases of significant contribution, reinforcing the idea of merit-based credit. Regular reviews of publication data, along with ways to address authorship concerns confidentially, can help reduce honorary authorship and maintain accountability in hierarchical research teams.

## Research Agenda

To examine how formal leadership relates to authorship, studies should evaluate leadership styles (e.g., supervisory versus hands-on), resource control (such as PI status, grant funding, lab size, and budget authority), and institutional context (like unit size and funding); record contributorship roles (e.g., CRediT-type statements) to confirm who contributed what; and analyze co-authorship networks (e.g., centrality) alongside inequality metrics (e.g., Gini). Conceptually, this work can draw on role theory and power-/social-exchange perspectives (leadership as resource control) and cumulative advantage (persistence of senior authorship once attained). A longitudinal or event-study design focused on leadership appointments, using matched non-leaders, would improve understanding of how formal authority influences author order, team composition, and inequality over time.

Moreover, comparing authorship patterns in Lithuanian medical academia with those in other countries could reveal whether similar trends exist elsewhere. Integrating these approaches, upcoming research can improve our understanding of how institutional power influences authorship practices.
